# Efficient differentiation of human embryonic stem cells to retinal pigment epithelium under defined conditions

**DOI:** 10.1186/s13287-021-02316-7

**Published:** 2021-04-21

**Authors:** Ioannis J. Limnios, Yu-Qian Chau, Stuart J. Skabo, Denver C. Surrao, Helen C. O’Neill

**Affiliations:** Clem Jones Centre for Regenerative Medicine, Bond University, Gold Coast, Queensland 4229 Australia

**Keywords:** Retinal pigment epithelium, Pluripotent stem cells, Differentiation, Small molecules, Age-related macular degeneration

## Abstract

**Abstract:**

Age-related macular degeneration (AMD) is a highly prevalent form of blindness caused by loss death of cells of the retinal pigment epithelium (RPE). Transplantation of pluripotent stem cell (PSC)-derived RPE cells is considered a promising therapy to regenerate cell function and vision.

**Objective:**

The objective of this study is to develop a rapid directed differentiation method for production of RPE cells from PSC which is rapid, efficient, and fully defined and produces cells suitable for clinical use.

**Design:**

A protocol for cell growth and differentiation from hESCs was developed to induce differentiation through screening small molecules which regulated a primary stage of differentiation to the eyefield progenitor, and then, a subsequent set of molecules to drive differentiation to RPE cells. Methods for cell plating and maintenance have been optimized to give a homogeneous population of cells in a short 14-day period, followed by a procedure to support maturation of cell function.

**Results:**

We show here the efficient production of RPE cells from human embryonic stem cells (hESCs) using small molecules in a feeder-free system using xeno-free/defined medium. Flow cytometry at day 14 showed ~ 90% of cells expressed the RPE markers MITF and PMEL17. Temporal gene analysis confirmed differentiation through defined cell intermediates. Mature hESC-RPE cell monolayers exhibited key morphological, molecular, and functional characteristics of the endogenous RPE.

**Conclusion:**

This study identifies a novel cell differentiation process for rapid and efficient production of retinal RPE cells directly from hESCs. The described protocol has utility for clinical-grade cell production for human therapy to treat AMD.

**Supplementary Information:**

The online version contains supplementary material available at 10.1186/s13287-021-02316-7.

## Introduction

Age-related macular degeneration (AMD) is the most common cause of blindness in people aged over 60 years and affects approximately 200 million people worldwide [1]. While the molecular mechanisms of AMD remain unclear, growing evidence suggests that metabolic stress [2] and inflammation [3], in conjunction with risk factors such as age, genetics, diet, and smoking, play major roles in disease development [4].

The retinal pigment epithelium (RPE) is the basal layer of the retina and is required for the survival and function of photoreceptors. In advanced stages of AMD, death and/or dysfunction of RPE cells in the macula trigger photoreceptor degeneration, resulting in loss of central vision [5].

Early-stage AMD presents as drusen deposits underneath the RPE, which then advances to one of two major forms: wet and dry AMD [6]. Wet AMD accounts for 10% of cases and is characterized by the uncontrolled growth of sub-retinal capillaries that can invade through the Bruch’s membrane to rapidly destroy the RPE and associated photoreceptors [7]. Standard treatment for wet AMD is disruption of angiogenic pathways by monthly intravitreal injections that target the VEGF signaling pathways [8]. Although anti-VEGF therapy can delay the advancement of wet AMD, 10–15% of patients is non-responsive or develops tachyphylaxis [9]. Dry AMD accounts for 90% of AMD cases and is associated with geographic atrophy of the RPE and retinal degeneration in the macula [10]. There is currently no effective treatment for dry AMD; however, disease onset can be delayed by dietary supplementation [11].

Cell therapies for AMD aim to halt or reverse disease progression by sub-retinal transplantation of healthy RPE cells into the macula to prevent photoreceptor loss and restore function [12–14]. Proof-of-concept for RPE transplantation has been established in congenital and injury models of retinal degeneration in animals, particularly in the Royal College of Surgeons (RCS) rat [15, 16]. However, human transplantation studies in AMD using autologous and non-autologous RPE cells have produced variable and short-lived benefits [17]. Critical barriers to clinical translation include patient disease state and progression, surgical complexity (particularly in autologous transplantation), and the limited availability and expansion of healthy, functional, and immune compatible RPE cells [18].

Human pluripotent stem cells (hPSCs) are a potentially unlimited source of RPE cells. Key advantages of hPSC-derived cell production include genome selection or editing before differentiation and extensive in vitro and in vivo quality control of hPSC-derived cell products prior to human transplantation [19]. Presently, clinical trials are testing the safety and efficacy of hPSC-RPE cell transplantation in AMD patients [20].

To date, most clinical trials generate hPSC-RPE cells by spontaneous differentiation to minimize the risk of patient exposure to xenogens [20]. However, spontaneous differentiation is labor intensive, inefficient, and highly variable across hPSC lines [21]. Manual enrichment can be improved using RPE-specific antibodies [22], or lipoprotein uptake [23], followed by cell sorting using flow cytometry and expansion of cell batches in culture. Nevertheless, cell batch expansion must be limited in order to maintain hPSC-RPE cell quality and function for cell therapy [24].

Directed differentiation protocols can significantly increase yields in hPSC-RPE cell production [25–29]. However, the most efficient protocols remain incompatible with cell therapy due to the use of undefined animal products [28, 29]. Thus, there remains a need for a robust and efficient protocol for hPSC-RPE clinical-grade cell.

Here, we hypothesize that an efficient hESC-RPE cell differentiation protocol can be developed using small molecules that recapitulate critical signaling events during RPE cell development in vivo. By first determining an optimal signaling strategy, we then adapt the protocol to xeno-free defined conditions to demonstrate proof-of-concept for high differentiation efficiencies using conditions that may be used for commercial scale production of cells for therapy.

## Materials and methods

### Human embryonic stem cell culture

MEL-1 hESCs (NIH Registry #0139, passage 29–40; StemCore, Brisbane, QLD, Australia) were maintained in mTeSR medium (StemCell Technologies, Vancouver, BC, Canada) on hESC-qualified Matrigel (MG: Corning Life Science, Lowell, CA, USA). Cultures were fed daily and passaged every 5 to 7 days using collagenase IV (1 mg/mL; Gibco, Carlsbad, CA, USA).

### Primary fetal RPE cell culture

Human fetal RPE (Φ-RPE) cells (ScienCell Research Labs: HRPEpiC #6540; Australian Biosearch, Wangarra, WA, Australia) were thawed and seeded at 50,000 cells/cm^2^ in xeno-free/defined RPE Expansion Medium (XFD-REM) (Table S1) on growth factor reduced Matrigel (GFR-MG: BD Biosciences, San Jose, CA, USA) for one passage. For maturation, cells were dissociated using TrypLE™ Express (Life Technologies, Melbourne, VIC, Australia) at day 7 and seeded at 150,000 cells/cm^2^ on GFR-MG in XFD-REM (Table S1).

### Induction of hESC-RPE differentiation through inhibition of TGFβ and WNT signaling

hESC colony cultures were incubated with the Rho Kinase inhibitor Y-27632 (Rock Inhibitor, 10 μM: Sigma-Aldrich, St Louis, MO, USA) for 1 h and dissociated to single cells using TrypLE™ Express. Cell aggregates (1000–1500 cells) were formed using the AggreWell™400 system (StemCell Technologies) and cultured in Basal Differentiation Media (BDM) with Rock inhibitor (Table S1). For differentiation, aggregates were cultured in BDM supplemented with combinations of SB43218 (SB: 10 μM), CKI-7 (CKI: 5 μM) and nicotinamide (NIC: 10 mM) (all Sigma-Aldrich). After 6 days, embryoid bodies (EBs) were plated on GFR-MG-coated plates in BDM.

At day 8, expanding EBs were dissociated with collagenase IV (1 mg/mL) and re-plated as clumps of 200 to 500 cells on GFR-MG-coated 24-well dishes. Cells were then cultured in BDM without factors and refed every 2 to 3 days until day 38.

### Directed two-stage differentiation of hESC-RPE cells using small molecules

hESCs were dissociated and plated on GFR-MG-coated 10 cm plates at ~ 300,000 cells/cm^2^ in mTeSR with 10 μM Rock Inhibitor. After reaching 90% confluence, medium was changed to xeno-free/defined (XFD)-BDM (Table S1) supplemented with SB (10 μM), LDN193189 (LDN: 100 nM) (Miltentyi Biotec, Sydney, NSW, Australia), CKI (5 μM), and NIC (10 mM). At day 5, differentiating cells were treated with Rock Inhibitor for 1 h, dissociated, and formed into 3D aggregates (~ 1000 cells) termed Anterior Neural Ectodermal Bodies (ANEBs) using Aggrewells™. ANEBs were cultured in suspension for 12 h and plated on to 6-well plates coated with natural mouse laminin (Thermo Fisher Scientific, Waltham, MA, USA) in XFD-BDM with primary differentiation factors for a further 24 h. Secondary differentiation was initiated on day 7 by changing medium to XFD-BDM, supplemented with either CHIR99021 (CHIR: 3 μM) and/or IDE-2 (IDE: 250 nM) or LDN + SB (LDN/SB) until day 16.

### Small molecule exclusion assay

To determine the contribution of small molecules to hESC-RPE cell differentiation at day 14, hESCs were prepared as above, seeded at 100,000 cells/cm^2^, and grown for 3 days or until ~ 90% confluent. Differentiation was initiated as described above with slight modifications, using combinations of SB, LDN, CKI, and NIC. Aggregates (~ 1200 cells/body) were formed at day 2 and cultured in suspension until day 6 in their respective primary differentiation conditions. At day 6, aggregates were plated on Growth Factor-Reduced Matrigel (GFR-MG; StemCell Technologies) coated 6-well plates. Twelve hours later, each primary differentiation condition was changed to one of several secondary differentiation conditions with combinations of IDE, CHIR, and NIC and cultured until day 14. Optimal differentiation conditions were determined through morphological analysis of outgrowths at day 14 and expressed as percentage of total cells with early RPE morphology. From day 14, cultures were matured in XFD-BDM (Table S1) without factors until the appearance of mature, pigmented, polygonal hESC-RPE cells (day 28).

### Xeno-free/defined hESC-RPE cell differentiation

For differentiation under XFD conditions, hESCs were adapted to mTeSR-E8 medium and then passaged onto Vitronectin-XF (VXF; StemCell Technologies) with ReLSR (StemCell Technologies) and differentiated as ANEBs in XFD-BDM supplemented with SB/LDN/CKI/NIC (primary differentiation) and IDE/CHIR (secondary differentiation) on VXF-coated plates.

### Expansion and maturation of hESC-RPE cells to epithelial monolayers

Differentiated hESC-RPE cells were expanded by whole-dish passage using TrypLE™ Express and plated on either GFR-MG or VXF at 50,000–100,000 cells/cm^2^ in XFD-REM. For maturation of passaged hESC-RPE cells, cells were plated on to GFR-MG or VXF at 150,000 cells/cm^2^ in XF-REM supplemented with 10% XFSR and Rock Inhibitor for 4 days or until confluent. For maturation, the concentration of XFSR was first reduced to 5% and then to 2% by day 7, constituting XFD-RPE maturation medium (XFD-RMM). In some experiments, cells were grown on GFR-MG-coated culture plates or polyethylene (PET) Transwell® membranes (Corning, NY, USA).

### Cell cryopreservation

hESC cultures were cryopreserved at 50–70% confluence. Briefly, hESCs were treated with Y-27632 (10 μM, Rock Inhibitor) for 1 h, cut into small pieces of ~ 100–300 cells, and resuspended in 1 ml of pre-chilled XF cryopreservation media (40%XFSR, 50%F12, 10%DMSO) or L7™hPSC Cryosolution (Lonza, Basel, Switzerland). Cells were transferred to a freezing container (Nalgene, Rochester, NY, USA) and slow frozen at −80 °C overnight before long-term storage in liquid nitrogen.

For hESC-RPE cells and human fetal RPEs, 70–80% confluent cultures were dissociated using TrypLE™ Express, counted and cryopreserved at 5 × 10^6^ cells/mL in XFD cryopreservation media (40% XFSR, 50% DMEM, 10% DMSO), and slow frozen, as described above.

### Photomicroscopy

Cell images were captured using Olympus CKX41 Microscope (Olympus Corporation, Centre Valley, PA, USA) equipped with a Nikon DS-Fil-L2 camera (Nikon Instruments Inc., Minato City, Tokyo, Japan).

### Gene expression analysis

Total RNA was extracted from cells using PureZOL™ RNA Isolation Reagent (BioRad, Hercules, CA, USA) according to the manufacturer’s instructions. RNA concentration and A260:A280 ratio were quantified using a NanoDrop 2000c (Thermo Fisher Scientific). One μg of RNA was reverse transcribed using the iScript™ cDNA synthesis kit (BioRad), and quantitative PCR analysis (qPCR) carried out with SsoFast™ EvaGreen® Supermix (BioRad) using a CFX96 Touch Real-Time PCR machine (BioRad). qPCR experiments were performed in technical triplicate for each RNA sample. Data was analyzed using the comparative threshold cycle (CT) method with GAPDH or B-ACTIN as an endogenous control. Primers used in this study (Integrated DNA Technologies, Iowa, USA) are listed in Supplemental Materials.

### Immunofluorescence

hESC-RPE cells grown on GFR-MG or VXF were fixed with 4% paraformaldehyde/phosphate-buffered saline (1x PBS) (Sigma-Aldrich) for 10 min at room temperature, then permeabilized with 0.1% TritonX-100 (Roche, Indianapolis, IN, USA) in PBS for 3 min. Samples were then incubated with primary antibody (1:40–1:1000 dilution) in blocking buffer composed of 3% bovine serum albumin (BSA) (Sigma-Aldrich) in PBS for 90 min at room temperature. Unconjugated primary antibodies were washed and incubated with conjugated secondary antibodies (1:500; Invitrogen) for 30 min in the dark at room temperature. Cell nuclei were co-stained using Hoechst 33342 (1:1000; Life Technologies). F-actin was stained with Alexa-Fluor-488-conjugated Phalloidin (Phalloidin-488) (1:40; Life Technologies) for 30 min in the dark at room temperature. Images were captured using a Nikon C1 Confocal microscope (Nikon Instruments Inc., Melville, NY, USA). Primary and secondary antibodies used in this study are listed in Supplemental Materials.

### Flow cytometry

Dissociated cells were fixed in 4% paraformaldehyde in PBS, centrifuged (4 °C, 300 g, 5 min), and washed in staining medium (PBS/2% XFSR). For intracellular marker studies, samples were permeabilized with 0.2% Triton X-100/PBS and washed as described above. Cells (~ 10^6^) were resuspended in staining medium containing fluorochrome-conjugated primary antibody or isotype control antibody for 30 min at 4 °C. Cells were washed three times in staining medium (4 °C, 300 g, 5 min). Unconjugated primary antibodies were stained with fluorochrome-conjugated secondary antibodies for 30 min at 4 °C and washed as described above. Labeled cells were analyzed using a FACSVERSE™ flow cytometer (Becton Dickinson Biosciences, Franklin Lakes, NJ, USA). Data analysis was performed using the FlowJo® software package (Ashland, OR, USA). Primary, secondary, and isotype-control antibodies used in this study are listed in Supplemental Materials (Table S2).

### Scanning electron microscopy (SEM)

hESC-RPE cells cultured on GFR-MG were washed twice with PBS and fixed in 3% glutaraldehyde (Sigma-Aldrich) in PBS for 24 h and then dehydrated through increasing concentrations of ethanol in PBS (25%, 45%, 55%, 65%, 75%, 85%, 90%, 95%, and 100%), followed by rinsing twice with hexamethyldisilazane (HMDS, Sigma-Aldrich). Samples were air-dried overnight and mounted on aluminum stubs and gold-coated using a gold-coater sputter (Jeol, Tokyo, Japan). Images were captured with a Neoscope JCM-5000 (Jeol) bench-top scanning electron microscope.

### Transepithelial electrical resistance (TEER)

hESC-RPE cells were seeded at 1.5 × 10^5^ cells/cm^2^ onto PET Transwell® membranes (Corning) coated with GFR-MG and cultured for 6 weeks in XFD-RMM (Table S1). Weekly TEER values (Ω•cm^2^) were obtained using an STX2 electrode (World Precision Instruments, Sarasota, FL, USA) from 3 replicate cultures each measured in triplicate. Background resistance was measured in triplicate equivalent wells containing no cells. Final values were obtained by subtracting background resistance from cell culture readings.

### Cytokine ELISA

The polarized secretion of vascular endothelial growth factor-A (VEGF-A) and pigment epithelium-derived factor (PEDF) from mature hESC-RPE cells was determined by ELISA. Briefly, medium from the apical and basal compartments of hESC-RPE cells cultured in Transwells was collected after 48 h from triplicate culture wells. The concentrations of VEGF-A and PEDF were determined using the human VEGF-A ELISA Kit (Boster Biological Technologies Ltd., Pleasanton, CA, USA) and human PEDF ELISA Kit (Wuhan EIab Science Ltd., Wuhan, China) with a microplate reader (Modulus™ II Microplate Multimode Reader; Turner Biosystems, Sunnyvale, CA, USA), according to manufacturer’s instructions.

### Phagocytosis

The procedure to measure receptor-mediated phagocytosis of hESC-RPE cells in cultures was adapted from a previous study [30]. Briefly, 1-μm fluorescent microspheres (Invitrogen) were incubated with VXF (5 μg/ml) in 20 mM HEPES binding buffer (Thermo Fisher Scientific) for 1 h at 37 °C. The reaction was then blocked with 0.1% BSA in HEPES binding buffer for 1 h at 37 °C. Controls were fluorescent microspheres treated with HEPES binding buffer containing 0.1% BSA for 2 h. Mature hESC-RPE cells were then incubated with 10^6^ spheres per cell and cultured for 24 h at 37 °C. Excess beads were removed by washing 5 times in PBS. Nuclei were stained using Hoechst 33342 (1:1000). Imaging was performed using an EVOS FL microscope (Thermo Fisher Scientific). To confirm internalization of microspheres, samples were fixed for 10 min in 4% paraformaldehyde in PBS, rinsed three times in PBS, followed by nuclear staining with Hoechst 33342 and F-Actin staining with Alexa-Fluor-488-conjugated Phalloidin (1:40) for 30 mins in the dark at room temperature. Images were taken using a Nikon C1 Confocal microscope.

### Karyotypic analysis

Karyotype analysis of Passage 11 hESC-RPE cells was performed by a commercial genotyping service (Sullivan Nicolaides Pathology, Brisbane, QLD, Australia). G-band analysis (> 40 metaphase spreads) was performed at a resolution of at least 400 bands per haploid set.

### Statistical analyses

Biological replicates were expressed as mean ± standard deviation (S.D). Significance was assessed using the Student’s two-sided *t* test (*p* = 0.05).

## Results

### Differentiation of hESCs to hESC-RPE cells

Initially, RPE cell differentiation from the MEL-1 hESC line was confirmed using protocols adapted from previous studies. Briefly, hESCs were cultured as embryoid bodies in the presence of SB and CKI-7 [26, 31], with and without NIC [25, 26, 31] (Fig. S1a). By day 5, pluripotent markers (OCT4 and SSEA4) were downregulated and early retinal lineage markers (PAX6 and RAX) were upregulated under both conditions. However, marker changes occurred earlier and at higher levels in the presence of NIC (Fig. S1b) [20–22]. Culture of cells from day 6 to day 32 in the absence of factors resulted in the appearance of foci containing cells with early RPE morphology, with approximately 3-fold more foci in the presence of NIC (Fig. S1c, d). These data indicate that the MEL-1 line is not defective for RPE differentiation using established methods [31] and that differentiation can be enhanced by early exposure to NIC [25].

To confirm hESC-RPE cell identity, day 21 foci were manually selected, dissociated, and replated in BDM alongside fetal RPE controls showing classical RPE morphology after 1 week [32] (Fig. S1e & f). By day 32, both cultures formed RPE fluid-filled domes [33] (Fig. S1g), and hESC-RPE cell cultures expressed RPE65 and ZO-1 consistent with previous studies [33, 34] (Fig. S1h), and forming apical microvilli by day 36 (Fig. S1i). These data confirm that morphology is a useful and non-invasive tool to quantify early stage RPE differentiation in culture, supported by the subsequent expression of RPE markers and mature cell characteristics at later stages.

### High efficiency of small molecule-directed hESC-RPE cell differentiation

**Fig. 1:**
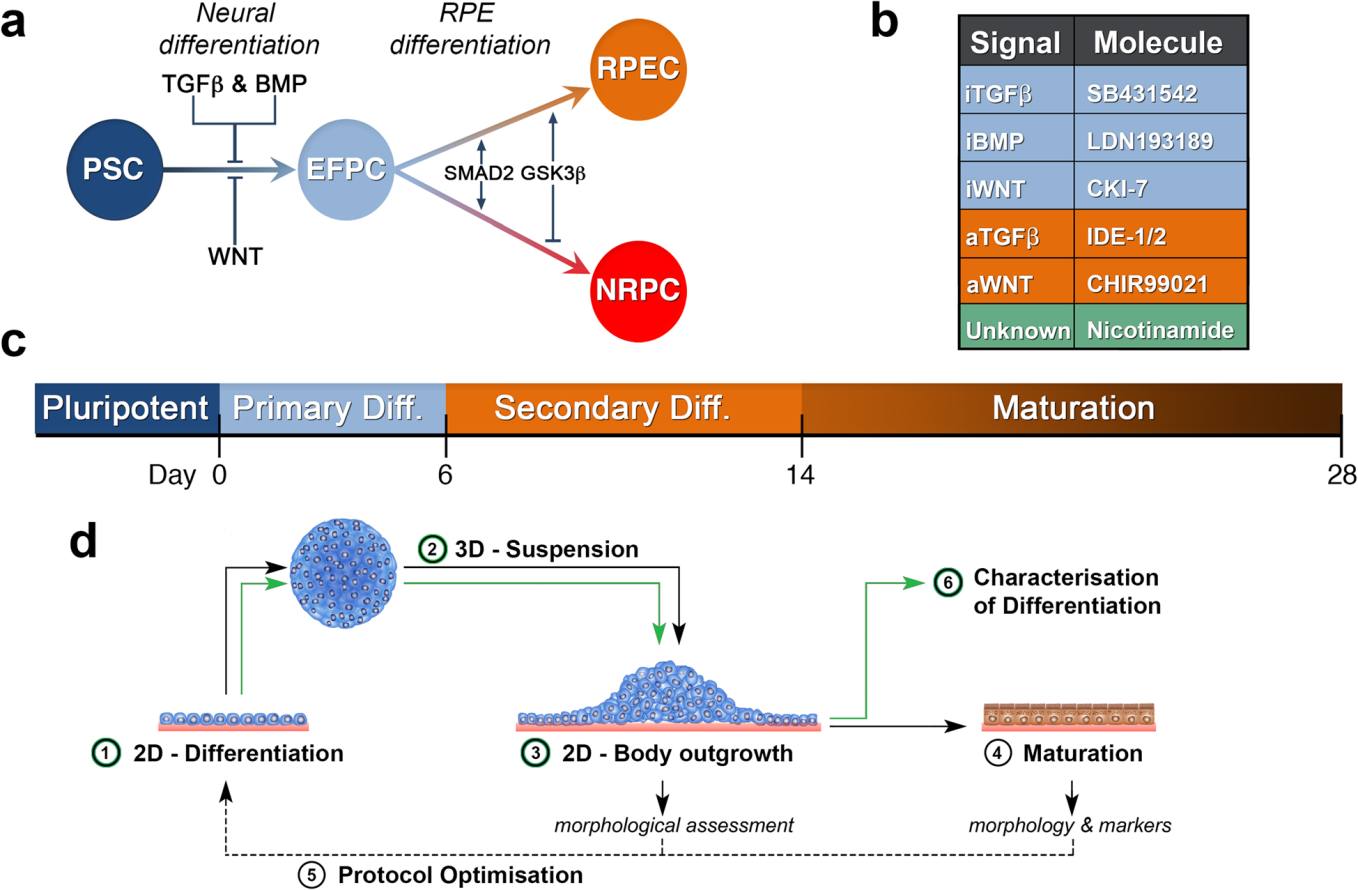
A schematic representation for differentiation of PSCs towards retinal cells. **a** Known signaling pathways and regulators in the differentiation of PSCs to RPE cells (RPECs) and neural retinal progenitor cells (NRPCs) via formation of a common eyefield progenitor cell (EFPC). **b** List of small molecules for development of a differentiation protocol as substitute endogenous signals: antagonists (blue), agonists (orange). **c** Timeline for in vitro differentiation of hESC-RPE cells. **d** Schematic showing stages in hESC differentiation. Stage 1: initiation of differentiation under adherent or 2D conditions. Stage 2: 3D aggregation of differentiating cells forming embryoid bodies. Stage 3: continued differentiation through plating cell bodies for outgrowth of differentiating cells. Stage 4: maturation of RPE cells with identification of cells through morphology and marker expression. Stage 5: optimization of the differentiation protocol through body size, timing of signaling switch and time of plating. Stage 6: characterization of differentiation through gene expression and cell functionality

In developing a model for RPE differentiation, we hypothesized that efficient hESC-RPE cell differentiation could be achieved by mimicking key RPE developmental pathways using small molecules (Fig. 1a). RPE development involves two major steps: differentiation of pluripotent cells to the anterior neural ectoderm (ANE)/eyefield (EF), followed by specification of EF cells to the RPE [35]. In a two-stage differentiation model, hESCs were first differentiated using inhibition of dual SMAD signaling (SB and LDN) [36] and WNT (CKI ± NIC) to generate ANE/EF cells [31, 37] (Fig. 1b, c), followed by activation of Activin (IDE) [38] and WNT signaling pathways (CHIR) [39] (Fig. 1b). To minimize the effects of limited diffusion within aggregates, primary differentiation was initiated under 2D conditions for 2 days prior to formation of 3D aggregates (Fig. 1d). For secondary differentiation, ANE bodies (ANEBs) were plated as aggregates to minimize the effects of seeding density on RPE differentiation efficiencies [40] (Fig. 1d).

hESC-RPE cells emerged between days 8 and 12 in all cultures treated with SB/LDN/CKI ± NIC (primary) followed by CHIR or CHIR/IDE, but not IDE alone (secondary) (Fig. S2a). Consistent with early experiments and previous studies, the use of NIC during primary differentiation enhanced final RPE cell differentiation (Fig. S2b). Continued suspension culture of ANEBs in the absence of CHIR after day 14 resulted in clusters of heavily pigmented cells with identifiable melanosomes by 4 weeks (Fig. S2c). These results suggest that the two-stage small molecule differentiation protocol can result in higher levels of hESC-RPE cell differentiation by day 14.

### Small molecule exclusion reveals optimal requirements for efficient differentiation

**Fig. 2:**
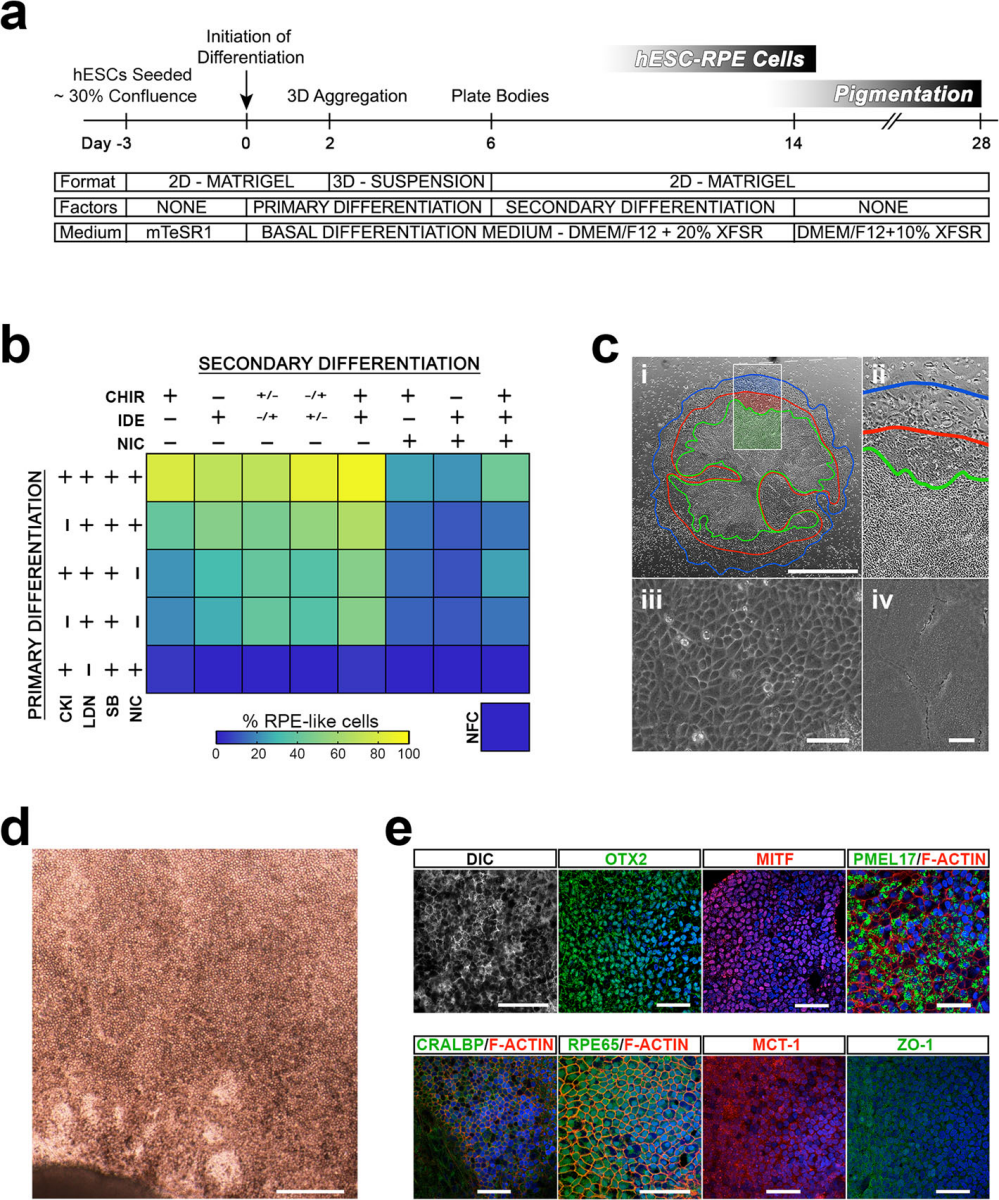
Optimization of differentiation by small molecule exclusion assay. The procedure for plating cells, managing embryoid bodies, and then replating cells was optimized to obtain maximal outgrowth of differentiated cells by 14 days. **a** Schematic of cell differentiation, including details of physical manipulation, timing, substrate, medium and signaling. **b** Schematic of different factors and their combinations to develop an optimized differentiation protocol. (+) present, (−) absent, (+/−) days 7–9, (−/+) days 10–14. Morphological analysis was used to calculate outgrowth of cells with early RPE morphology as percentage of total cells. NFC no factor controls. **c** Cells grown under the best small molecule conditions of CKI/LDN/SB/NIC (primary) followed by CHIR/ IDE (secondary). A representative body is shown at day 16 with contours demarcating zones of cells with different morphology. Scale = 1000 μm. The green zone identifies cells with classical RPE cell morphology, the red zone demarcates outgrowth of more fibroblastic cells and the blue zone shows the leading edge of the body (i, ii). Phase contrast microscopy shows cells at day 16 in the green zone with typical early RPE cell morphology (iii). Scale = 50 μm. SEM shows immature hESC-RPE cells without microvilli (iv). Scale = 2 μm. **d** After 28 days of growth, cell monolayers under phase microscopy reflect uniformly pigmented, polygonal RPE cells. Scale = 200 μm. **e** Differential interference contrast (DIC) microscopy shows dark pigmentation of in vitro matured hESC-RPE cells. Immunofluorescent staining shows expression of key RPE cell transcription factors (OTX2 and MITF), pre-melanocyte marker (PMEL17), visual cycle proteins (CRALBP and RPE65), and mature RPE cell markers (MCT-1 and ZO-1). Phalloidin stained F-actin identifies cell boundaries. Hoescht33342 was used to stain nuclei. Scale = 50 μm

To gain greater insight into hESC-RPE cell differentiation, we investigated the contribution of small molecules to the process through systematic exclusion (Fig. 2a, b). Since previous studies have used NIC for prolonged periods during RPE differentiation [25, 41], we also tested the effects of NIC during secondary differentiation. At day 14, the early hESC-RPE cell morphology was measured in ANEB outgrowths as a proportion of total cells (Fig. 2b).

Across all secondary differentiation conditions, final differentiation outcomes were relatively higher in outgrowths generated from SB/LDB/CKI/NIC (primary differentiation) ANEBS (Fig. 2b). As expected, exclusion of LDN in primary differentiation resulted in the complete loss of hESC-RPE cells under most secondary differentiation conditions (Fig. 2b), while exclusion of CKI and/or NIC in primary differentiation reduced differentiation by day 14 (Fig. 2b). With respect to secondary conditions, the exclusion of CHIR resulted in the greatest decrease in final RPE differentiation levels. While IDE alone did not enhance final RPE differentiation, the combination of CHIR/IDE resulted in more robust differentiation relative to all primary conditions. Unexpectedly, we found that the addition of NIC during secondary differentiation had a negative effect on hESC-RPE cell differentiation (Fig. 2b). The optimal signaling strategy for hESC-RPE cell differentiation (SB/LDN/CKI/NIC + IDE/CHIR) resulted in outgrowths, comprised almost entirely of hESC-RPE cells (Fig. 2c).

Cells differentiated under optimal primary and secondary conditions were cultured without factors between days 14 and 28. The absence of factors between days 14 and 28 gave rise to pigmented, polygonal cells (Fig. 2c) that stained for early RPE markers (OTX2, MITF, PMEL17) and for late RPE markers (CRALBP, RPE65) (Fig. 2d). Weak apical staining of MTC-1 and ZO-1 at tight junctions suggested incomplete maturation (Fig. 2e). Taken together, these data support our signaling model for hESC-RPE differentiation within 2 weeks.

### A protocol for efficient reproducible differentiation

**Fig. 3:**
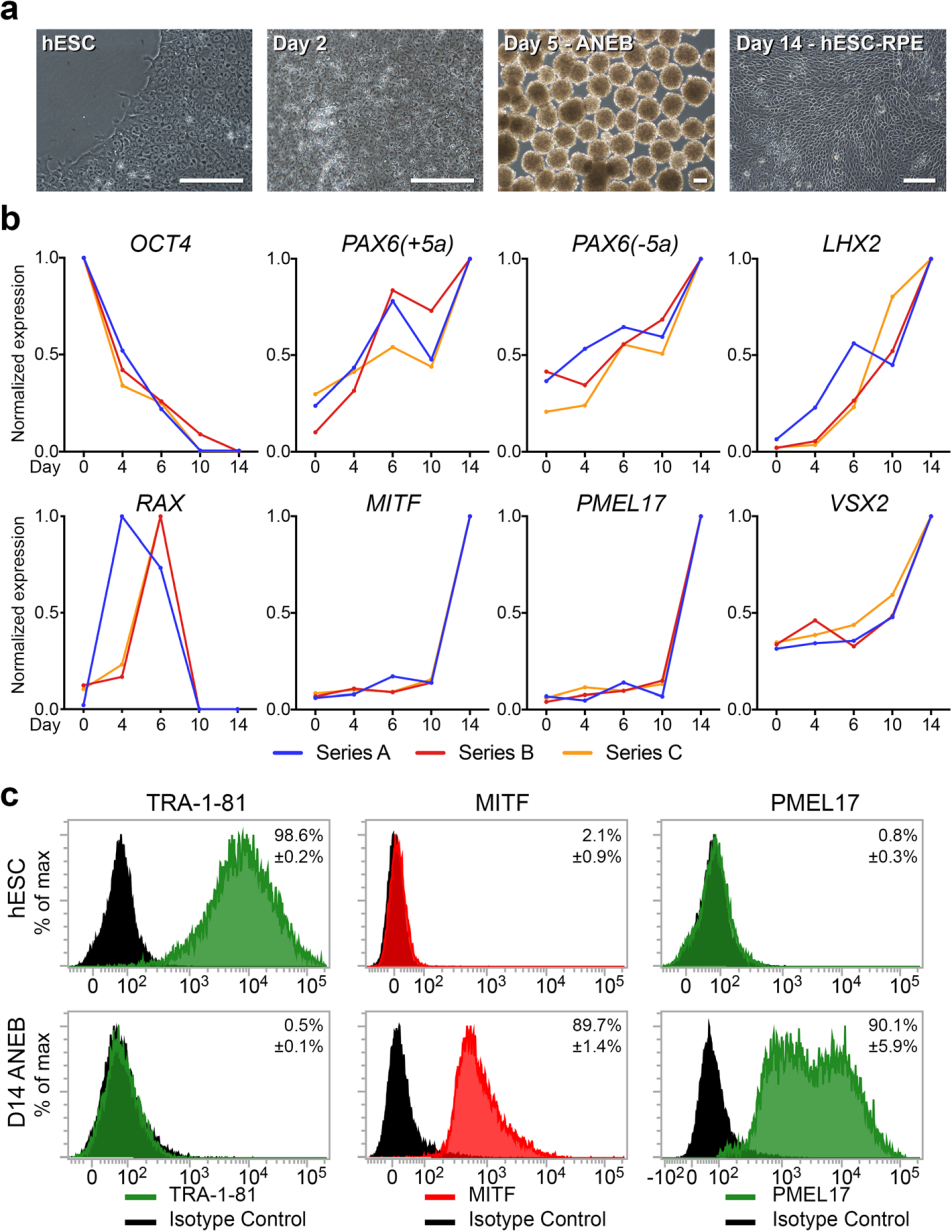
Evidence of in vitro differentiation. **a** Phase contrast microscopy shows key morphological changes: Human embryonic stem cell (hESC: MEL-1 line) morphology prior to differentiation, formation of differentiating cell monolayers (day 2), and anterior neuroectoderm bodies (ANEBs) (day 5). Plated ANEBs produce expanding sheets of hESC-RPE cell monolayers in the outgrowth by day 14. Scale = 100 μm. **b** Gene expression over the 14-day differentiation period was measured by qPCR in relation to *GAPDH* and normalized to maximum expression for each gene over the time period. Normalized expression profiles indicate loss of hESCs (*OCT4*), upregulation of neuroectoderm [*PAX6(+5a)*, *PAX6(−5a)*, and *LHX2*], eyefield formation (*RAX*), and differentiation of RPE cells (*MITF* and *PMEL17*). Contaminating neural retinal cell formation was monitored through expression of *VSX2*. Series A, B, C reflect independent time course experiments and data points reflect the average of technical triplicates. **c** Flow cytometry indicates expression of TRA-1-81, MITF, and PMEL17 by hESCs at day 0, and differentiation hESC-RPE (ANEBs) at day 14. Data reflect mean + S.D. (*n* = 3). For each marker, significant differences are detected in the staining of hESCs versus day 14 ANEBs (*p* < 0.0001)

Repeated differentiation experiments on Matrigel resulted in reproducible and consistent morphologies at distinct timepoints (Fig. 3a). Reproducibility of differentiation was also reflected in gene expression analysis measured by qPCR between days 0 and 14. Transcriptional changes during this time revealed stepwise hESC-RPE cell differentiation via ANE/EF cell intermediates (Fig. 3b). In particular, the partial (~ 50%) downregulation of *OCT4* by day 4, combined with upregulation of *PAX6(−5a)* (retinal specific gene splice form) and *LHX2*, plus the transient upregulation of *RAX* between days 4 and 6, is consistent with eyefield differentiation [42, 43] (Fig. 3b). The developmental competence of ANE/EF cells in day 6 cultures was further confirmed through photoreceptor differentiation under alternative secondary conditions, resulting in rhodopsin+/recoverin+ cells by day 21 (unpublished data). Upregulation of *MITF* and *PMEL17* between days 10 and 14 indicated RPE differentiation, while upregulation of *VSX2* indicates some non-specific differentiation to the neural retinal lineage (Fig. 3b). These data indicate that RPE differentiation has occurred via an anterior neuroectodermal/eyefield fate in our system.

Finally, we quantified hESC-RPE cell differentiation efficiencies at day 14 using samples from the triplicate experiments used for gene expression analysis. hESC cultures expressed TRA-1-81 (98.6% ± 0.2%), but very little MITF (2.1% ± 0.9%) or PMEL17 (0.8% ± 0.3%) (Fig. 3c). At day 14, TRA-1-81 was downregulated (0.5 ± 0.11%), while a high proportion of cells were positive for MITF (89.7 ± 1.44%, *n* = 3) and PMEL17 (90.1 ± 5.9%, *n* = 3) (Fig. 3c).

We then adapted the protocol to complete xeno-free/defined conditions to demonstrate the potential for clinical application. All reagents remained the same except for the use of the human recombinant substrate VXF to replace Matrigel. Prior to differentiation, hESCs were adapted to mTeSR-E8 and cultured on Vitronectin-XF (Fig. S3 a, b) and then differentiated under previously optimized conditions. Consistent with our previous data, day 14 ANEB outgrowths contained high proportions of cells with early hESC-RPE cell morphology (Fig. S3c). Quantification of differentiation efficiencies by flow cytometric analysis showed PMEL17 expression in ~ 85% of cells (Fig. S3d). Continued culture from day 14 to day 28 in XFD-BDM resulted in ANEB outgrowths with lightly pigmented, cobblestone monolayers (Fig. S3e) and several fluid-filled domes (not shown). Immunofluorescence at day 28 showed cytoplasmic expression of PMEL17, with ZO-1 expression localized at tight junctions (Fig. S3f). Overall, these data demonstrate the simple adaptation of hESC-RPE cell differentiation to xeno-free/defined conditions by changing the substrate from Matrigel to Vitronectin-XF.

Taken together, these data confirm the robust differentiation of hESC-RPE cells along known developmental cell fates at high efficiency and can be translated to a GMP (Good Manufacturing Practice) process with minimal changes in reagents.

### Stability of expanded hESC-RPE cells

**Fig. 4:**
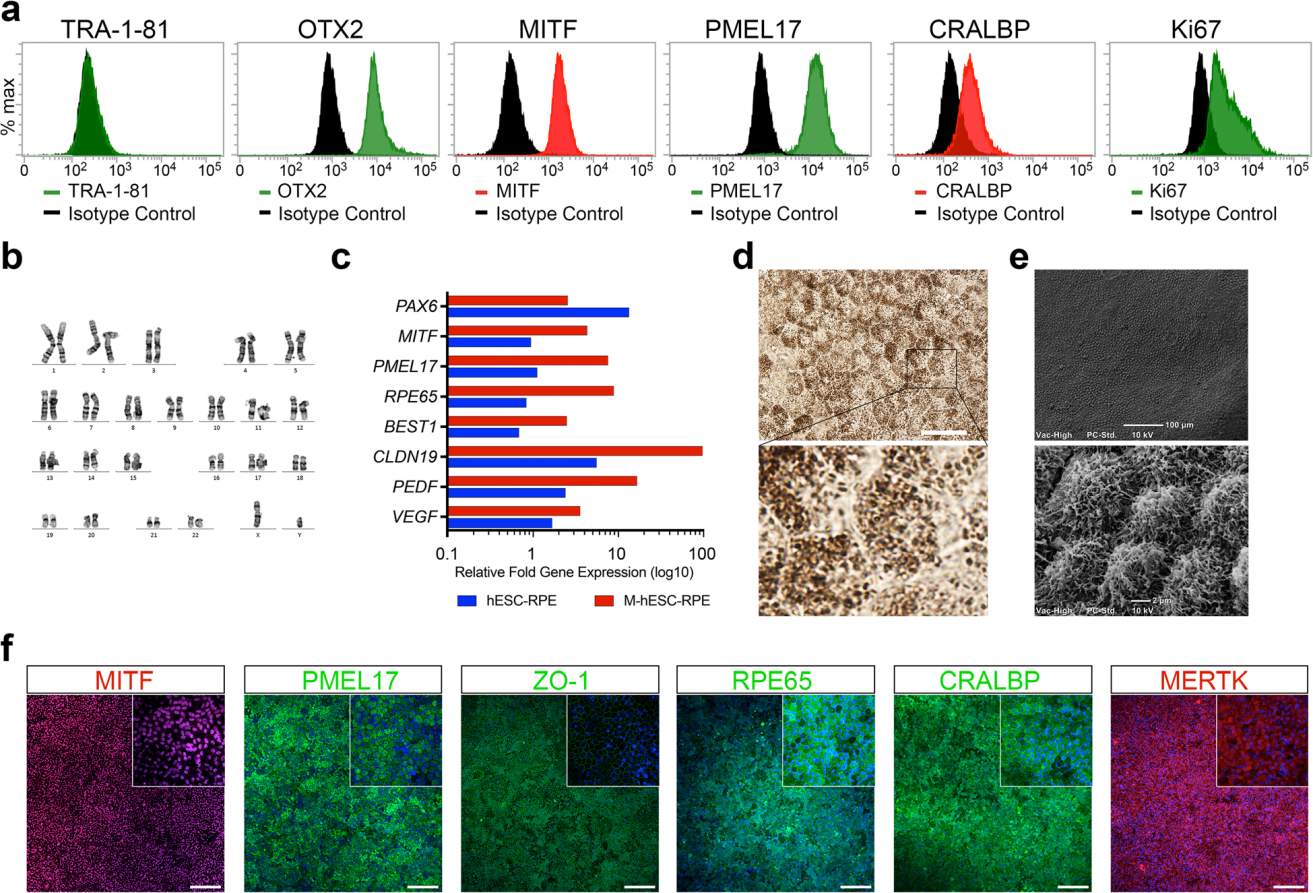
Cultured hESC-RPE cells maintain identity following expansion. **a** Flow cytometric analysis of RPE markers (OTX2, MITF, PMEL17, and CRABLP), the proliferation marker (Ki67) and pluripotent hESC marker (TRA-1-81) in sub-confluent, actively dividing cells in hESC-RPE cell cultures. **b** Karyotypic analysis of hESC-RPE cells at passage 11. **c** Gene expression analysis comparing immature hESC-RPE (I-hESC-RPE) cells with maturing hESC-RPE (M-hESC-RPE) cells expressed relative to human fetal RPE cells. **d** Re-acquisition of pigmented, cobblestone morphology in hESC-RPE cell monolayers after 4 weeks under optimized maturation conditions. Scale = 100 μm. **e** Scanning electron microscopic images of mature hESC-RPE cells with developed apical microvilli. Scale = (top) 100 μm, (bottom) 2 μm. **f** Confocal immunofluorescent detection of MITF, PMEL17, ZO-1, RPE65, CRALBP, and MERTK in matured hESC-RPE cell monolayers

RPE cells have limited potential for in vitro expansion and lose markers of cell fate over extended culture [24]. We therefore investigated the ability of hESC-RPE cells to maintain RPE markers after prolonged expansion. Flow cytometry showed maintenance of RPE (OTX2, MITF, PMEL17, and CRALBP) and proliferation (Ki67) markers at passage 8 (Fig. 4a). The lack of detectable TRA-1-81 expression indicated the absence of dedifferentiated cells or the persistence of pluripotent cells in long-term cultures. Furthermore, karyotypic analysis at passage 11 showed cells retained a normal karyotype (Fig. 4b). No evidence of epithelial-to-mesenchymal transition was seen by passage 11.

Further analysis of hESC-RPE cell maturation by qRT-PCR showed distinct differences in the expression of early and late RPE genes between immature (day 14) and mature (P6) hESC-RPE cells (Fig. 4c). Compared to immature cells, mature cells showed downregulation of the early retinal differentiation marker (*PAX6*), maintenance of RPE fate genes (*MITF*), and upregulation of genes required for RPE function, pigmentation (*PMEL17*), the visual cycle (*BEST-1*, *RPE65*), cytokine production (*PEDF*, *VEGF-A*), and tight junction formation (*CLDN19*) (Fig. 4c). The gene expression data indicated hESC-RPE cell maturation at the transcriptional level. Further evidence of maturation could also be seen in the re-pigmentation of cells and the development of apical microvilli (Fig. 4d, e). Finally, immunofluorescence from antibody staining showed highly uniform expression of key RPE markers (MITF, PMEL17, ZO-1, RPE65, CRALBP, and MERTK) (Fig. 4e).

### In vitro function of mature hESC-RPE cells on PET membranes

**Fig. 5:**
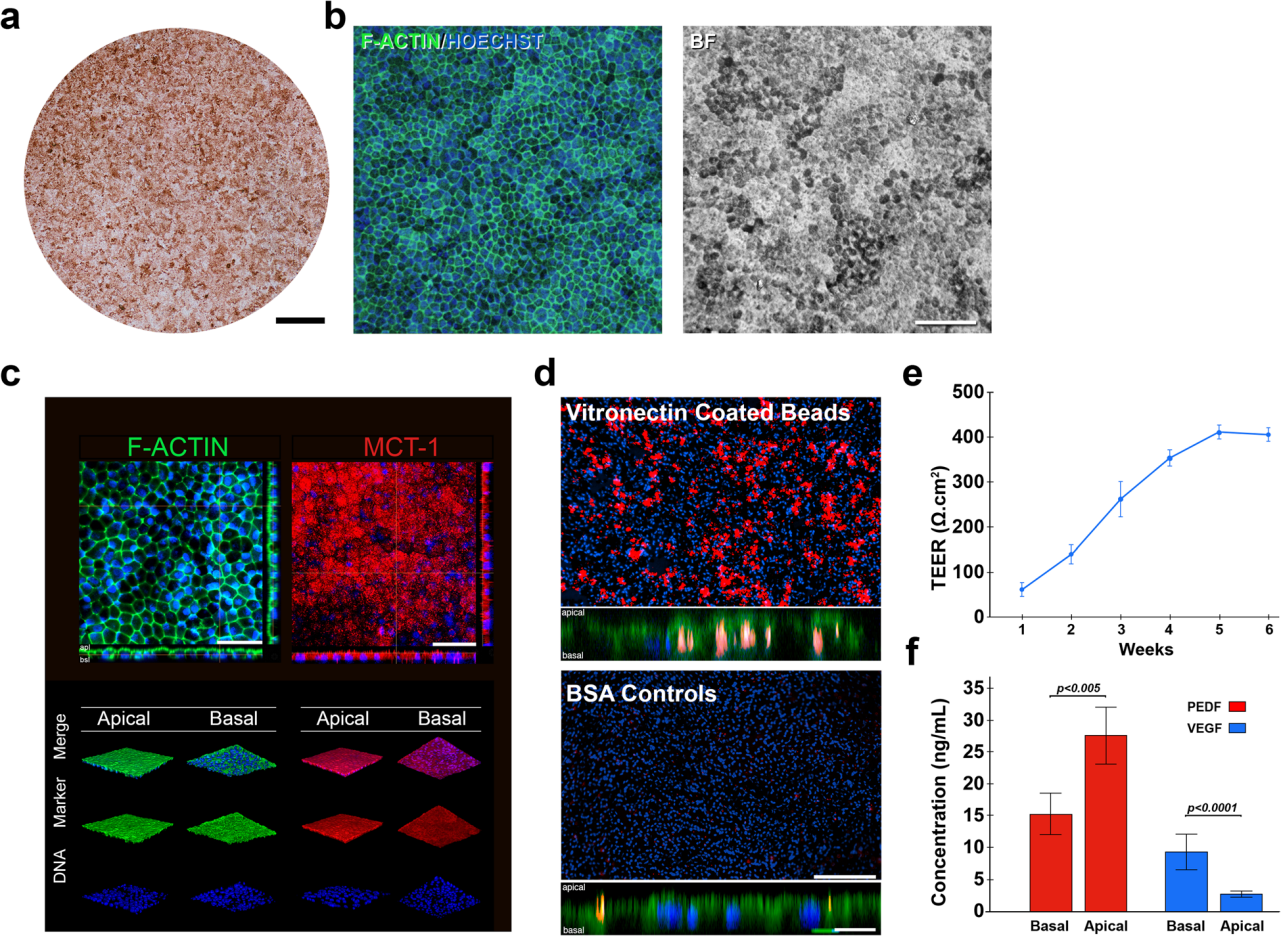
Mature cell characteristics. **a** Mature hESC-RPE cells grown on PET membranes at day 28. **b** Fluorescent detection of F-actin (phalloidin) on mature hESC-RPE monolayers (left) with DIC photomicroscopy (right). Scale = 100 μm. **c** Confocal *Z*-stack analysis of F-actin (Phalloidin) and MCT-1 in mature hESC-RPE monolayers. Scale = 50 μm. **d** Uptake of fluorescent beads coated with VXF or BSA (control) demonstrate receptor-mediated phagocytosis. Scale = 400 μm. *Z*-stack confocal microscopy shows internalization of beads, shown below cell membranes. Phalloidin staining of F-actin (green) shows apical membrane of hESC-RPE cells. Scale = 15 μm). **e** Measurement of transepithelial electrical resistance (TEER) over 6 weeks (*n* = 4). **f** Directional secretion of VEGF and PEDF from mature hESC-RPE cells (*n* = 3)

The literature suggests that an optimal cell therapy for AMD is a mature, polarized monolayer of hPSC-RPE cells delivered on a supportive scaffold [20]. We therefore created a prototype implant similar to that used in human clinical studies [44] in order to study maturation and function of cells grown on a membrane in vitro*.* Passage 11 hESC-RPE cells were seeded on polyethylene (PET) Transwell membranes in xeno-free/defined retinal maturation media (XFD-RMM: Table S1) and characterized at various timepoints. Photomicroscopy at day 28 revealed the formation of homogeneous RPE monolayer reminiscent of the polarized native epithelium, with well-organized cobblestone architecture and pigmentation (Fig. 5a, b). Monolayers expressed F-ACTIN and MCT-1 localized to tight junctions and apical microvilli, respectively (Fig. 5c).

Mature hESC-RPE cell monolayers were then tested for critical functions of the native RPE. Phagocytosis of photoreceptor outer segments is a highly specific function of RPE cells mediated in part via the vitronectin receptor αvβ5 which is required for outer segment phagocytosis [45]. Following 24-h exposure, hESC-RPE cell monolayers showed preferential binding and uptake of vitronectin-coated beads compared to BSA controls (Fig. 5d). Transepithelial electrical resistance (was measured to test barrier function across week 1 through week 6. TEER values steadily rose until week 4 (~ 400 Ω × cm^2^) and remained constant through to week 6 (Fig. 5e). Finally, hESC-RPE cell monolayers secreted PEDF and VEGF-A in a polarized manner and at physiologically relevant levels, consistent with normal RPE function (Fig. 5f).

Taken together, these data suggest that methods developed here for generation, expansion, and maturation of hESC-RPE cells result in phenotypically and functionally stable cells that maintain the functional properties of the native RPE required for cell therapy up to at least 11 passages.

## Discussion

The clinical translation of hPSC technology promises to transform the treatment of AMD over the coming decade. To date, the production of hPSC-RPE cells for use in human trials has mostly depended on spontaneous differentiation [20]. However, treatment of large patient numbers will require much more efficient differentiation protocols to generate large numbers of homogeneous functional cells that cannot easily be generated by the protocols used for clinical trials to date. In this study, we have developed a rapid directed protocol to generate hESC-RPE cells at high efficiency using small molecules, under feeder-free, xeno-free, and chemically defined conditions.

The efficiency of our protocol was shown to be consistently high and reproducible, indicating the robustness of the method. While the differentiation efficiencies obtained here are similar to previous studies [28, 29], we show fast and efficient differentiation in the complete absence of cytokines and under xeno-free/defined conditions. Other studies working towards clinical grade cell production have claimed high efficiencies using XFD media and substrates; however, they did not show subjective measures of differentiation efficiency prior to passage and expansion [46]. Similarly, a recent method by Regent et al. (2019) describes a differentiation process achieved with the sequential use of three factors (Nicotinimide, Activin A, and CHIR99021). However, this method requires a 6-week process and no data on yield was provided immediately after differentiation and prior to dissociation-based selection [47].

Our signaling model for in vitro RPE cell differentiation is based on the sequence of signals that drive pluripotent cells of the human blastocyst to the eyefield and, subsequently, the RPE. Small molecule inhibition of the dual SMAD pathways (SB/LDN) and WNT signaling pathways (CKI) follows a highly conserved developmental mechanism [48, 49] and is consistent with both the “default” model [50] and the “active” model of anterior neural differentiation [51]. As in previous studies, we found dual SMAD inhibition was necessary for efficient neural induction within a week [36, 37, 46], while WNT inhibition [44] and NIC were required for the anterior specification of neuroectoderm to form the eyefield.

The timing of differentiation from the eyefield to early RPE cells is in accordance with the development of the optic primordium (eyefield) at Carnegie Stage 10 (day 22) [52] and the first appearance of melanin granules in the RPE at stage 13 (day 32) [53], which is approximately 10 days. Our protocol is also similar to previous in vitro studies showing efficient RPE differentiation from hESCs by 14 days [28], suggesting that the rate of differentiation in our system is highly similar to both in vivo and in vitro development of RPE cells.

WNT inhibition with CKI and NIC were both required during primary differentiation (days 0–6) for optimal differentiation by day 14, indicating an additive effect of both via a common target. Although NIC has been used in several hESC-RPE cell differentiation protocols [25, 28, 29, 41], the proposed mechanism of activity was previously attributed to increased cell survival during neural differentiation [28, 41]. However, in this study, we noted higher levels of cell death under conditions using NIC (Fig. 2b). A recent study identified a possible mechanism for the redundancy of NIC, which we note here, involving a role for NIC in inhibition of casine kinase 1 [54], also consistent with our differentiation model.

Secondary differentiation was primarily dependent on WNT activation using CHIR and is consistent with specification of the eyefield to the RPE via activation of *MITF* [42, 55, 56]. For any given primary differentiation condition, secondary differentiation with IDE resulted in higher consistency of final RPE differentiation, but at lower efficiency than CHIR alone. Optimal differentiation required the combined use of both CHIR and IDE. Since IDE does not fully mimic the Activin-like signaling required for RPE development using Activin A, this finding will require further investigation.

The addition of NIC during secondary differentiation lowered final differentiation outcomes when used in combination with CHIR and IDE, consistent with its proposed role in inhibition of WNT signaling via inhibition of casine kinase 1 [54]. These findings suggest that existing protocols using NIC may benefit from the deliberate exclusion of NIC after the eyefield stage [25, 41].

Gene expression profiling across day 0 to day 14 revealed that hESC-RPE cell differentiation occurs via a defined intermediate population with characteristic expression of eyefield markers [28]. This is consistent with the existence of a multipotent eyefield progenitor cell (EFPC) as shown in Fig. 1. This differentiation model can then be as a basis to generate and identify an EFPC and to divert differentiation along the neural retinal lineage, with possible generation of photoreceptors or ganglion cells. Consistent with this hypothesis, our own experiments have found that differentiating hESCs are competent to form either RPE cells or photoreceptors depending on treatment after primary differentiation (unpublished data).

Mature hESC-RPE cells generated in this study form homogeneous polarized monolayers in culture dishes and when grown on a planar membrane, such that cells retain key morphological, molecular, and functional hallmarks of the native RPE. After cryopreservation and further expansion to passage 11, hESC-RPE cells retained the ability to form highly homogeneous monolayers of polarized epithelium, while maintaining RPE marker expression and function need for cell therapy. These results at passage 11 are therefore significant, showing that our cells were capable of reaching molecular and functional maturity after expansion and cryopreservation, which is an important consideration in view of clinical and commercial scale production and storage.

Oncogenic transformation of cells is a concern when cells are intended for human therapy. We have observed no unusual cell growth during tissue culture, but instead, routinely see contact inhibition in hESC-RPE cells upon reaching confluence. In the course of this work, we have also conducted in vivo transplantation studies into vision-impaired pigmented RCS-p (Royal College of Surgeons) rats and can report an absence of tumor formation at 1 month post-transplantation of cells into the sub-retinal space (data in preparation). However, the safety of hESC-RPE cells will need to be established leading up to clinical trial and this will require high coverage genomic sequencing as well as extensive gene expression studies to identify any changes in the genome or expression of tumor and anti-apoptotic genes. Such a detailed study is anticipated ahead of production of GMP-grade cells for pre-clinical and clinical studies similar to those carried out in other pre-clinical studies [57].

## Conclusion

The hESC-RPE cell differentiation protocol presented in this study addresses the need for efficiency under xeno-free/defined conditions. Adaptation of the protocol to a strictly 2D adherent format represents a critical step towards a manufacturing process. To date, the procedure has been applied to three distinct hESC lines and is under test on hIPSC lines. The method forms the ground work for development of a robotic manufacturing process to generate cells for banking and later transplantation.

## Supplementary Information

**Additional file 1: Fig. S1.** Initial protocol used for differentiation of hESCs to RPE cells based on embryoid body (EB) formation.

**Additional file 2: Fig. S2.** Primary and secondary differentiation conditions designed to drive hESCs to RPEs within 6 Days.

**Additional file 3: Fig. S3.** Adaptation of the hESC-RPE cell differentiation protocol to xeno-free conditions.

**Additional file 4: Table S1.** Media compositions.

**Additional file 5: Table S2.** List of antibodies used in the study.

**Additional file 6: Table S3.** List of qPCR primers used in the study.

## Data Availability

All data will be made available upon reasonable request to the corresponding author.

## Abbreviations

ANE: Anterior neural ectoderm
ANEB: Anterior neural ectodermal bodies
AMD: Age-related macular degeneration
BDM: Basal differentiation medium
CHIR: CHIR99021
CKI: CKI-7
EB: Embryoid body
EF: Eyefield
EFPC: Eyefield progenitor cell
hESC: Human embryonic stem cells
GFR: Growth factor reduced
hPSC: Human pluripotent stem cells
LDN: LDN193189
MG: Matrigel
NIC: Nicotimamide
PEDF: Pigment epithelium-derived factor
PET: Polyethylene terephthalate
RCS: Royal College of Surgeons
RPE: Retinal pigment epithelium
REM: Retinal expansion medium
RMM: Retinal maturation medium
SB: SB43218
SEM: Scanning electron microscopy
TEER: Transepithelial electrical resistance
VEGF-A: Vascular endothelial growth factor A
VXF: Vitronectin XF
XFD: Xeno-free/defined
