# Supplementary material for: Efficient differentiation of human embryonic stem cells to retinal pigment epithelium under defined conditions
Source: Stem Cell Res Ther. 2021 Apr 21;12:248. doi: 10.1186/s13287-021-02316-7 (PMC8058973; doi:10.1186/s13287-021-02316-7)
Supplement: Supplementary file 1 — Additional file 1: Fig. S1. Initial protocol used for differentiation of hESCs to RPE cells based on embryoid body (EB) formation. [file 13287_2021_2316_MOESM1_ESM.docx]

**Fig. S1**


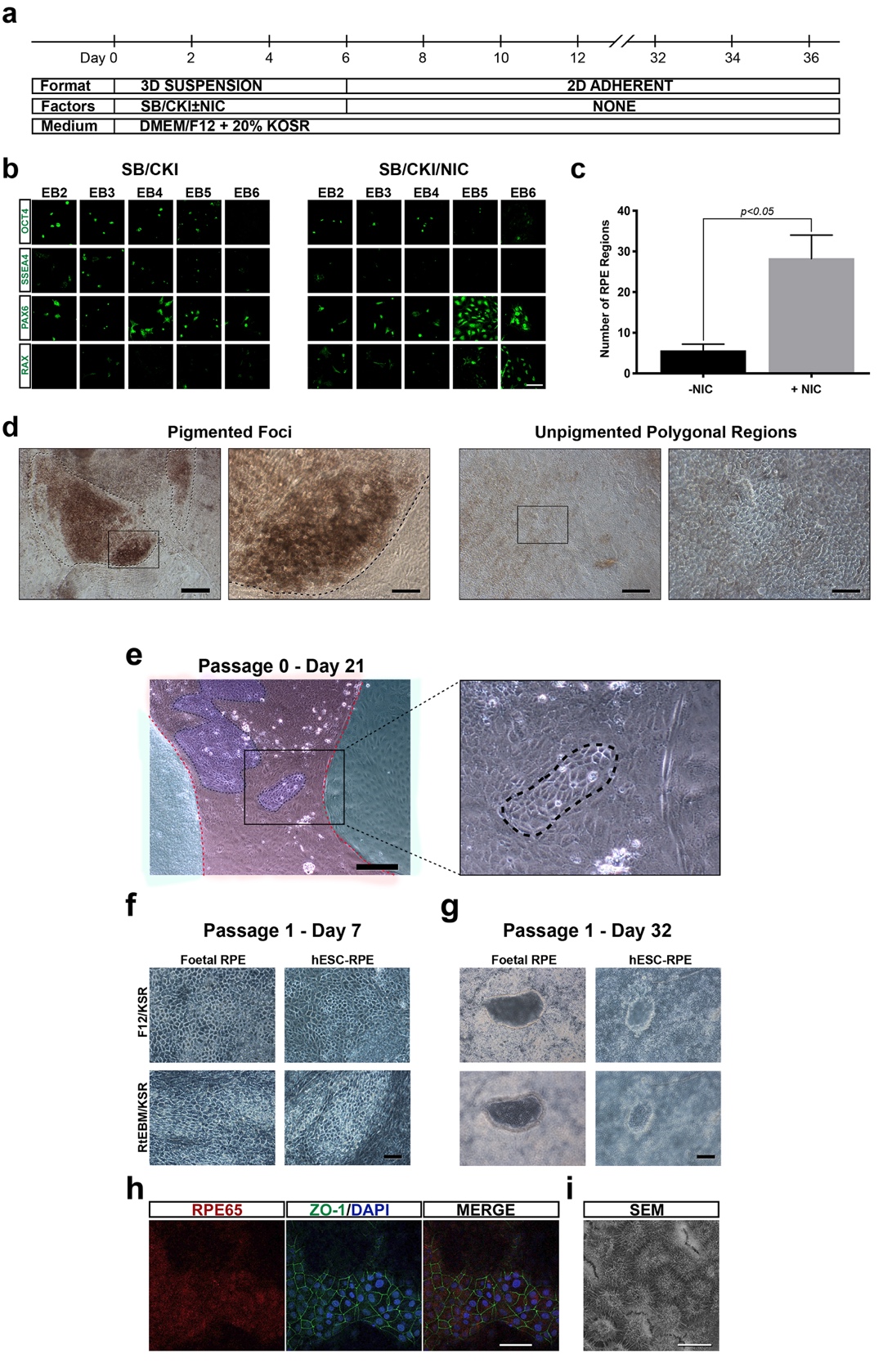


**Fig. S1** Initial protocol used for differentiation of hESCs to RPE cells based on embryoid body (EB) formation. a) Schematic representation for differentiation of MEL-1 hESCs to RPE cells through EB. After 6 days, EBs were dissociated with collagenase IV and seeded on to growth factor reduced-Matrigel. b) Cells obtained from EB samples across Days 2 to 6 stained for markers of pluripotency (SSEA4, OCT4) and early retinal differentiation markers (RAX, PAX6). Scale bar = 50μm. c) Number of RPE-like regions at Day 32 in SB/CKI and SB/CKI/ NIC cultures (mean ± S.D., n=3). d) Phase microscopy of hESC-RPE cells at 32 Days showing unpigmented polygonal cells and regions with sporadic pigmentation. Scale = 100μm. e) Emergence of hESC-RPE cells in differentiating cultures 21 days after cultures were established. Low and high magnification views are shown. Scale = 200μm. f) Comparative morphology of human foetal RPE cells and hESC-RPE cells at Day 7. Scale = 50μm. g) Comparative morphology of human foetal RPE cells and hESC-RPE cells at Day 32 showing development of polygonal morphology and fluid-filled domes. Scale = 200μm. h) Immunofluorescence detection of RPE65 and ZO-1 in hESC-RPE cells at Day 32. Scale = 40μm. i) Scanning electron microscopy (SEM) of hESC-RPE cells at Day 36 showing the development of apical microvilli. Scale = 20μm.
