# Supplementary material for: Efficient differentiation of human embryonic stem cells to retinal pigment epithelium under defined conditions
Source: Stem Cell Res Ther. 2021 Apr 21;12:248. doi: 10.1186/s13287-021-02316-7 (PMC8058973; doi:10.1186/s13287-021-02316-7)
Supplement: Supplementary file 2 — Additional file 2: Fig. S2. Primary and secondary differentiation conditions designed to drive hESCs to RPEs within 6 Days. [file 13287_2021_2316_MOESM2_ESM.docx]

**Fig. S2**


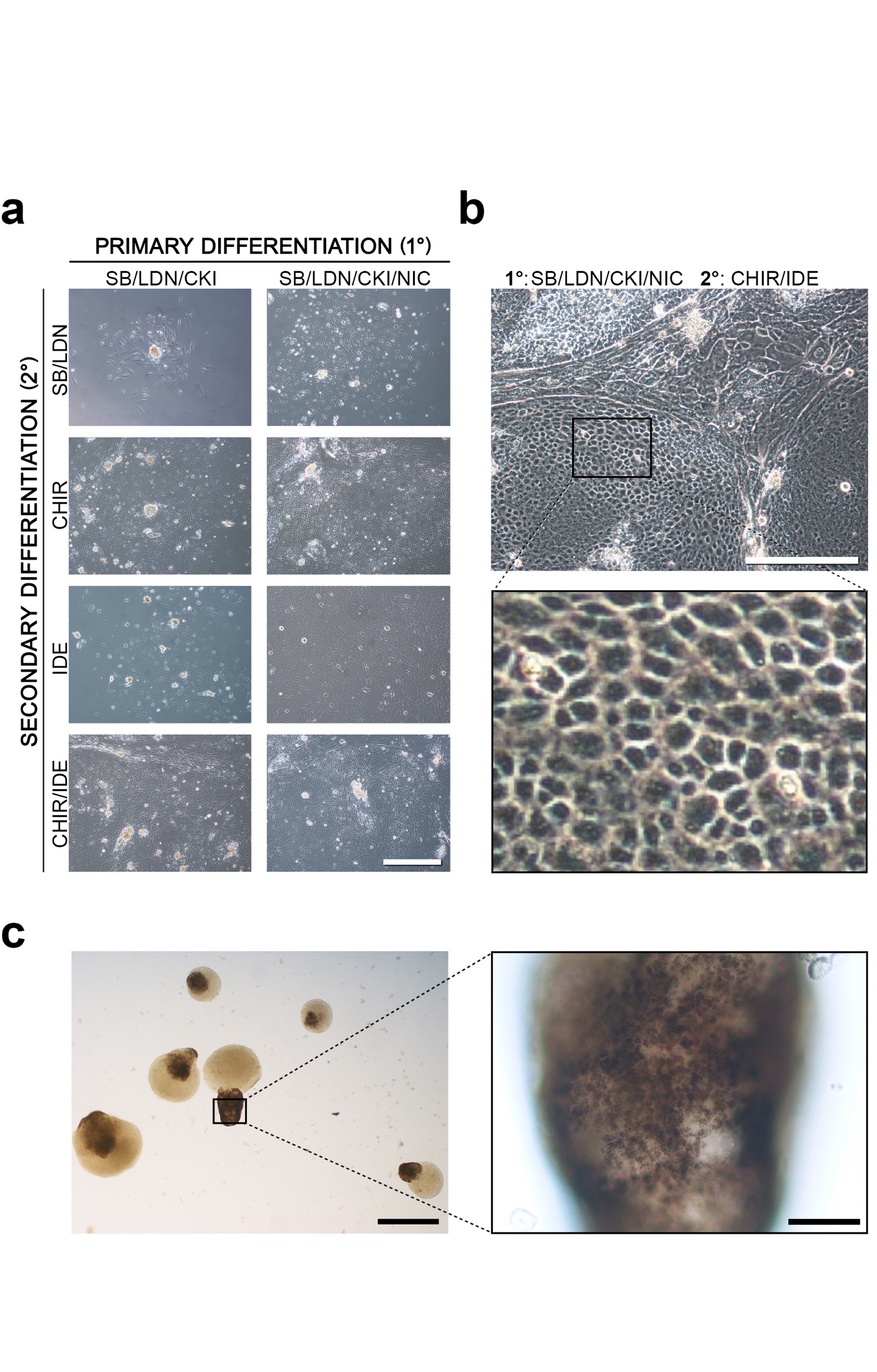


**Fig. S2** Primary and secondary differentiation conditions designed to drive hESCs to RPEs within 6 days. a) Phase contrast microscopy of hESC-RPE cells obtained under the best combination of small molecules for secondary differentiation by Day 14. The condition includes selected combinations of SB, LDN, CHIR and IDE. Scale = 200μm. b) High magnification image of hESC-RPE cells grown under the best secondary differentiation conditions. Scale = 200μm. c) Image of 3D aggregates in suspension cultures displaying pigmented regions at Day 28. Scale = 400μm. High magnification image depicting polygonal cells with granular pigmentation reminiscent of RPE cells. Scale = 100μm.
