# Supplementary material for: Efficient differentiation of human embryonic stem cells to retinal pigment epithelium under defined conditions
Source: Stem Cell Res Ther. 2021 Apr 21;12:248. doi: 10.1186/s13287-021-02316-7 (PMC8058973; doi:10.1186/s13287-021-02316-7)
Supplement: Supplementary file 3 — Additional file 3: Fig. S3. Adaptation of the hESC-RPE cell differentiation protocol to xeno-free conditions. [file 13287_2021_2316_MOESM3_ESM.docx]

**Fig. S3** ­­

**
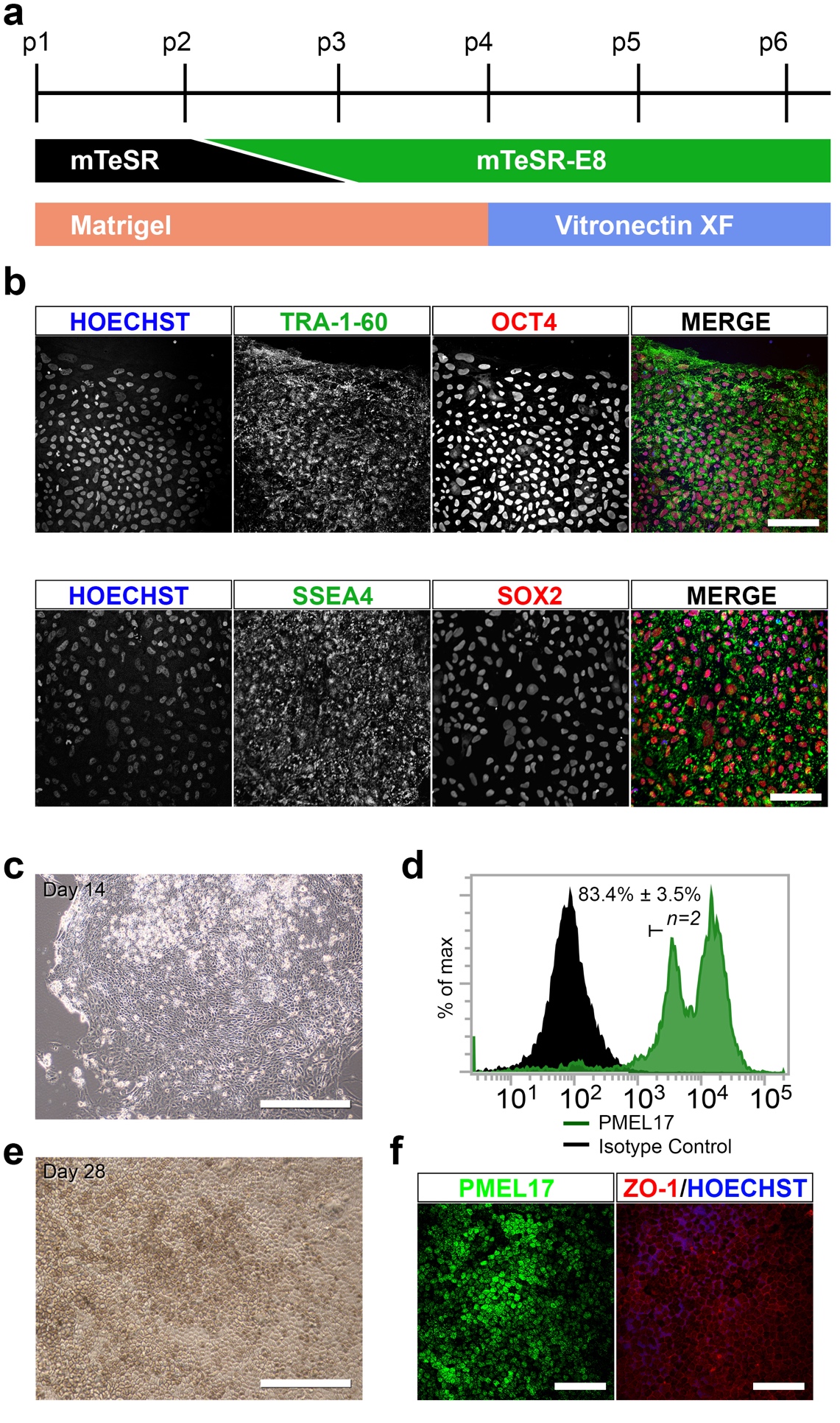
**

**Fig. S3** Adaptation of the hESC-RPE cell differentiation protocol to xeno-free conditions. a) A schematic showing the timeline for hESC adaptation to xeno-free (XF) culture conditions. b) Immunofluorescence detection of pluripotency markers OCT4, TRA-1-60, SOX2 and SSEA4 in hESCs grown under xeno-free/defined conditions with Hoechst 33342 staining of nuclei. Scale = 50μm. c) Following differentiation under the protocol developed here, xeno-free hESC-RPE cells appeared in ANEB outgrowths by Day 14. Scale = 100μm. d) Determination of differentiation efficiency at Day 14 by detection of PMEL17 using flow cytometry. e) Photomicrograph of hESC-RPE cells showing cobblestone morphology and pigmentation at Day 28. f) Confirmation of RPE markers PMEL17 and ZO-1 on maturing xeno-free hESC-RPE cells. Scale = 100μm.
