# Supplementary material for: Efficient differentiation of human embryonic stem cells to retinal pigment epithelium under defined conditions
Source: Stem Cell Res Ther. 2021 Apr 21;12:248. doi: 10.1186/s13287-021-02316-7 (PMC8058973; doi:10.1186/s13287-021-02316-7)
Supplement: Supplementary file 4 — Additional file 4: Table S1. Media compositions. [file 13287_2021_2316_MOESM4_ESM.pdf]

Table S1 - Media Formulations

1. Xeno-free/defined RPE Expansion Medium (XFD-REM) (20%)

Company	Product Full Name	Catalogue #	[Final]	For 100mL	For 500mL
Life Technologies	DMEM	11995073	76.9%	76.9mL	384.5mL
LifeTechnologies	KnockOut SR XenoFree CTS	12618-013	20%	20mL	100mL
Life Technologies	Sodium Pyruvate Solution 100 mM (100X)	11360-070	1mM	1mL	5mL
Life Technologies	Non-Essential Amino Acids Solution 10 mM (100X)	11140050	100μM	1mL	5mL
Life Technologies	Glutamax 200mM (100x)	35050-061	2mM	1mL	5mL
Sigma	β-Mercaptoethanol (1000x)	21985023	55μM	100μL	500μL

2. Xeno-free/defined RPE Expansion Medium (XFD-REM) (10%)

Company	Product Full Name	Catalogue #	[Final]	For 100mL	For 500mL
Life Technologies	DMEM	11995073	86.9%	86.9mL	434.5mL
LifeTechnologies	KnockOut SR XenoFree CTS	12618-013	10%	10mL	50mL
Life Technologies	Sodium Pyruvate Solution 100 mM (100X)	11360-070	1mM	1mL	5mL
Life Technologies	Non-Essential Amino Acids Solution 10 mM (100X)	11140050	100μM	1mL	5mL
Life Technologies	Glutamax 200mM (100x)	35050-061	2mM	1mL	5mL
Sigma	β-Mercaptoethanol (1000x)	21985023	55μM	100μL	500μL

3. Xeno-free/defined RPE Maturation Medium (XFD-RMM) (5%)

Company	Product Full Name	Catalogue #	[Final]	For 100mL	For 500mL
Life Technologies	DMEM	11995073	91.9%	91.9mL	459.5mL
LifeTechnologies	KnockOut SR XenoFree CTS	12618-013	5%	5mL	25mL
Life Technologies	Sodium Pyruvate Solution 100 mM (100X)	11360-070	1mM	1mL	5mL
Life Technologies	Non-Essential Amino Acids Solution 10 mM (100X)	11140050	100μM	1mL	5mL
Life Technologies	Glutamax 200mM (100x)	35050-061	2mM	1mL	5mL
Sigma	β-Mercaptoethanol (1000x)	21985023	55μM	100μL	500μL

4. 2% Xeno-free/defined RPE Maturation Medium (XFD-RMM) (2%)

Company	Product Full Name	Catalogue #	[Final]	For 100mL	For 500mL
Life Technologies	DMEM	11995073	94.9%	94.9mL	474.5mL
LifeTechnologies	KnockOut SR XenoFree CTS	12618-013	2%	2mL	10mL
Life Technologies	Sodium Pyruvate Solution 100 mM (100X)	11360-070	1mM	1mL	5mL
Life Technologies	Non-Essential Amino Acids Solution 10 mM (100X)	11140050	100μM	1mL	5mL
Life Technologies	Glutamax 200mM (100x)	35050-061	2mM	1mL	5mL
Sigma	β-Mercaptoethanol (1000x)	21985023	55μM	100μL	500μL

Table S2 - Antibodies I

Antibody	Host	Clone	Isotype	Conjugation	Supplier	Cat no.	Application
α-CRALBP	Mouse	B2	IgG2a	-	ABC	AB15051	IF/FC
α-Ki67	Rabbit	Polyclonal	IgG	-	ABC	AB833	FC
α-MCT-1	Mouse	Polyclonal	IgG	-	ABC	AB90582	IF
α-MITF	Mouse	C5	IgG1	-	ABC	AB12039	IF
α-MITF	Mouse	21D1418	IgG1	DyLight® 650	NVB	NB100-56561C	FC
α-MERTK	Rabbit	Polyclonal	IgG	-	INV	PA5-15028	IF
α-OCT4	Rabbit	Polyclonal	IgG	-	SGT	130-095-635	IF
α-PAX6	Rabbit	Polyclonal	IgG	-	ABC	AB5790	IF
α-PMEL17	Mouse	HMB45	IgG1k	DyLight® 488	NVB	NBP2-34638G	FC
α-PMEL17	Rabbit	EP4863(2)	IgG	-	ABC	AB137078	IF/FC
α-RAX	Mouse	4F4	IgG2ak	-	SIG	SAB1405061	IF/FC
α-RPE65	Mouse	Polyclonal	IgG	-	ABC	AB13826	IF
α-SOX2	Rabbit	Polyclonal	IgG	-	SGT	130-095-636	IF
α-SSEA-4	Mouse	MC-813-70	IgG3k	Alexa Fluor® 488	BDB	560308	IF
α-TRA-1-81	Mouse	TRA-1-81	IgMk	Alexa Fluor® 488	SCT	60065AD	FC
α-ZO-1	Mouse	ZO1-1A12	IgG1	Alexa Fluor® 488	INV	339188	IF
α-ZO-1	Rabbit	Polyclonal	IgG	-	INV	61-7300	IF

Application: Immunofluorescence (IF), Flow Cytometry (FC).

Antibody	Host	Clone	Conjugation	Supplier	Cat no.	Application
α-Mouse IgM	Goat	Polyclonal	FITC	SCT	A-10211	Secondary
α-Mouse IgG	Goat	Polyclonal	Alexa Fluor® 488	INV	A-11001	Secondary
α-Mouse IgG	Goat	Polyclonal	Alexa Fluor® 568	INV	A-11004	Secondary
α-Rabbit IgG	Goat	Polyclonal	Alexa Fluor® 488	INV	A-11008	Secondary
α-Rabbit IgG	Goat	Polyclonal	Alexa Fluor® 568	INV	A-11011	Secondary
α-Rabbit IgG	Goat	Polyclonal	Alexa Fluor® 647	INV	A-21244	Secondary
α-IgG	Rabbit	EPR25A	-	ABC	AB172730	Isotype
α-IgG1	Mouse	CT6	-	ABC	AB81216	Isotype
α-IgG1	Mouse	MOPC-21	DyLight® 650	NVB	NBP2-36577C	Isotype
α-IgG1k	Mouse	P3.6.2.8.1	DyLight® 488	NVB	NBP1-43319G	Isotype
α-IgG3k	Mouse	MG3-35	PE	MTB	130-095-620	Isotype
α-IgMk	Mouse	MM-30	DyLight® 488	SGT	09-0072	Isotype
α-IgMk	Mouse	MM-30	DyLight® 488	MTB	130-095-673	Isotype
α-IgMk	Mouse	MM-30	Alexa Fluor® 488	SCT	60069AD	Isotype
α-IgMk	Mouse	MM-30	PE	SCT	60069PE	Isotype

Supplier: Abcam (ABC), BD Biosciences (BDB), Invitrogen (INV), Miltenyi Biotech (MTB), Novus Biologicals (NVB), R&D Systems (R&D), Santa Cruz (SCZ), Sigma (SIG), Stemcell Technologies (SCT), Stemgent (SGT).
