# Supplementary material for: Efficient differentiation of human embryonic stem cells to retinal pigment epithelium under defined conditions
Source: Stem Cell Res Ther. 2021 Apr 21;12:248. doi: 10.1186/s13287-021-02316-7 (PMC8058973; doi:10.1186/s13287-021-02316-7)
Supplement: Supplementary file 5 — Additional file 5: Table S2. List of antibodies used in the study. [file 13287_2021_2316_MOESM5_ESM.pdf]

Table S2 - Antibodies I

| Antibody | Host | Clone | Isotype | Conjugation | Supplier | Cat no. | Application |
|---|---|---|---|---|---|---|---|
| α-CRALBP | Mouse | B2 | IgG2a | - | ABC | AB15051 | IF/FC |
| α-Ki67 | Rabbit | Polyclonal | IgG | - | ABC | AB833 | FC |
| α-MCT-1 | Mouse | Polyclonal | IgG | - | ABC | AB90582 | IF |
| α-MITF | Mouse | C5 | IgG1 | - | ABC | AB12039 | IF |
| α-MITF | Mouse | 21D1418 | IgG1 | DyLight® 650 | NVB | NB100-56561C | FC |
| α-MERTK | Rabbit | Polyclonal | IgG | - | INV | PA5-15028 | IF |
| α-OCT4 | Rabbit | Polyclonal | IgG | - | SGT | 130-095-635 | IF |
| α-PAX6 | Rabbit | Polyclonal | IgG | - | ABC | AB5790 | IF |
| α-PMEL17 | Mouse | HMB45 | IgG1k | DyLight® 488 | NVB | NBP2-34638G | FC |
| α-PMEL17 | Rabbit | EP4863(2) | IgG | - | ABC | AB137078 | IF/FC |
| α-RAX | Mouse | 4F4 | IgG2ak | - | SIG | SAB1405061 | IF/FC |
| α-RPE65 | Mouse | Polyclonal | IgG | - | ABC | AB13826 | IF |
| α-SOX2 | Rabbit | Polyclonal | IgG | - | SGT | 130-095-636 | IF |
| α-SSEA-4 | Mouse | MC-813-70 | IgG3k | Alexa Fluor® 488 | BDB | 560308 | IF |
| α-TRA-1-81 | Mouse | TRA-1-81 | IgMk | Alexa Fluor® 488 | SCT | 60065AD | FC |
| α-ZO-1 | Mouse | ZO1-1A12 | IgG1 | Alexa Fluor® 488 | INV | 339188 | IF |
| α-ZO-1 | Rabbit | Polyclonal | IgG | - | INV | 61-7300 | IF |

Application: Immunofluorescence (IF), Flow Cytometry (FC).

| Antibody | Host | Clone | Conjugation | Supplier | Cat no. | Application |
|---|---|---|---|---|---|---|
| α-Mouse IgM | Goat | Polyclonal | FITC | SCT | A-10211 | Secondary |
| α-Mouse IgG | Goat | Polyclonal | Alexa Fluor® 488 | INV | A-11001 | Secondary |
| α-Mouse IgG | Goat | Polyclonal | Alexa Fluor® 568 | INV | A-11004 | Secondary |
| α-Rabbit IgG | Goat | Polyclonal | Alexa Fluor® 488 | INV | A-11008 | Secondary |
| α-Rabbit IgG | Goat | Polyclonal | Alexa Fluor® 568 | INV | A-11011 | Secondary |
| α-Rabbit IgG | Goat | Polyclonal | Alexa Fluor® 647 | INV | A-21244 | Secondary |
| α-IgG | Rabbit | EPR25A | - | ABC | AB172730 | Isotype |
| α-IgG1 | Mouse | CT6 | - | ABC | AB81216 | Isotype |
| α-IgG1 | Mouse | MOPC-21 | DyLight® 650 | NVB | NBP2-36577C | Isotype |
| α-IgG1k | Mouse | P3.6.2.8.1 | DyLight® 488 | NVB | NBP1-43319G | Isotype |
| α-IgG3k | Mouse | MG3-35 | PE | MTB | 130-095-620 | Isotype |
| α-IgMk | Mouse | MM-30 | DyLight® 488 | SGT | 09-0072 | Isotype |
| α-IgMk | Mouse | MM-30 | DyLight® 488 | MTB | 130-095-673 | Isotype |
| α-IgMk | Mouse | MM-30 | Alexa Fluor® 488 | SCT | 60069AD | Isotype |
| α-IgMk | Mouse | MM-30 | PE | SCT | 60069PE | Isotype |

Supplier: Abcam (ABC), BD Biosciences (BDB), Invitrogen (INV), Miltenyi Biotech (MTB), Novus Biologicals (NVB), R&D Systems (R&D), Santa Cruz (SCZ), Sigma (SIG), Stemcell Technologies (SCT), Stemgent (SGT).
