# Supplementary material for: Efficient differentiation of human embryonic stem cells to retinal pigment epithelium under defined conditions
Source: Stem Cell Res Ther. 2021 Apr 21;12:248. doi: 10.1186/s13287-021-02316-7 (PMC8058973; doi:10.1186/s13287-021-02316-7)
Supplement: Supplementary file 6 — Additional file 6: Table S3. List of qPCR primers used in the study. [file 13287_2021_2316_MOESM6_ESM.pdf]

Table S3 - Primers

qPCR Primer Sequences

Transcript	Forward	Reverse
OCT4	AGTTTGTGCCAGGGTTTTTG	ACTTCACCTTCCCTCCAACC
PAX6 (+5a)	CTCGGTGGTGTCTTTGTCAAC	ACTTTTGCATCTGCATGGGTC
PAX6 (-5a)	AGTGAATCAGCTCGGTGGTGTCTT	TGCAGAATTCGGGAAATGTCGCAC
LHX2	TCGGGACTTGGTTTATCACCT	GCAAGCGGCAGTAGACCAG
RAX	GTTCGAGAAGTCCCACTACCCGGAC	CACTTAGCCCGTCGGTTCTGGAAC
MITF	TCACAACCTGATTGAACGAAGAA	ACTTTCGGATATAGTCCACGGAT
PMEL17	GTTGATGGCTGTGGTCCTTG	CAGTGACTGCTGCTATGTGG
RPE65	CAAGGCTGACACAGGCAAGA	TTGACGAGGCCCTGAAAAGA
BEST1	CTTGATGGAGCACCCAGAAGT	GCTTCATCCCTGTTTTCCAAGG
VEGF	GGAAACCTTCCTTCCACCCTT	TGTAGCCTGTCCCCTTCAAGA
PEDF	TTCAAAGTCCCCGTGAACAAG	GAGAGCCCGGTGAATGATGG
CLDN19	CTCAGCGTAGTTGGCATGAA	GAAGAACTCCTGGGTCACCA
VSX2	CGGCGACACAGGACAATCTT	TAGAGCCCATACTCCGCCAT
B-ACTIN	CAAGATCATTGCTCCTCCTGAG	TGCTTGCTGATCCACATCTG
GAPDH	AGAAGGCTGGGGCTCATTTG	AGGGGCCATCCACAGTCTTC
